# Chemical Contaminants and Food Quality

**DOI:** 10.3390/foods14132317

**Published:** 2025-06-30

**Authors:** Chang Li

**Affiliations:** State Key Laboratory of Food Science and Resources, Nanchang University, Nanchang 330047, China; lichang@ncu.edu.cn; Tel.: +86-791-88304447

## 1. Introduction

Ensuring the safety and integrity of the global food supply is a paramount challenge in an era of interconnected agricultural, industrial, and environmental systems [1]. Within the complex web, chemical contaminants emerge as pervasive threats. They quietly permeate to the source and end of our food chain through diverse and complex channels, including agricultural chemicals, such as pesticides, herbicides, and fertilizers widely used in farmlands [2]; environmental pollution like heavy metals and persistent organic pollutants caused by industrial production emissions and improper waste disposal [3,4]; hazardous by-products formed during food processing [5]; chemical substances in packaging materials that may migrate and interact in food products, especially plastics [6,7]; toxins that occur naturally in certain foods, such as mycotoxins and Marine biological toxins [8,9]; as well as intentional adulteration driven by economic interests, including illegal additives [10], all of which collectively contribute to the contamination sources.

These contaminants carry complex and multi-dimensional risks, whose hazards permeate the entire chain of food production, circulation, and consumption, significantly compromising food quality and safety. And the threats of contaminants to human health present a scenario of interwoven acute and chronic effects. Acute exposure may trigger symptoms, such as food poisoning and allergic reactions in a short period, while long-term chronic exposure can subtly damage human organ and system functions, such as carcinogenicity, endocrine disruption, and neurotoxicity [11,12]. Additionally, they jeopardize ecological sustainability via bioaccumulation processes and disruption of ecosystem equilibrium [13,14]. Therefore, these chemical contaminants, which range from persistent organic pollutants to acute natural toxins, transcend disciplinary boundaries, requiring integrated scientific inquiry to unravel their sources, fate in food matrices, and implications for human health.

This editorial introduces a Special Issue dedicated to advancing our understanding of chemical contaminants in foods, emphasizing cutting-edge research on detection methodologies, formation and migration mechanisms, and innovative control strategies. By synthesizing interdisciplinary insights, this collection aims to inform evidence-based policies and practices that safeguard food systems for present and future generations.

## 2. An Overview of Published Articles

Contribution 1 delved into the role of acrolein, a toxic compound that can be generated during food processing, in neurodegenerative diseases. The article demonstrated, through numerous in vitro and in vivo studies, a strong association between acrolein and neurodegenerative diseases such as ischemic stroke, Alzheimer’s disease, Parkinson’s disease, and Multiple Sclerosis. It indicated that endogenous acrolein produced by the disease and exogenous acrolein can trigger and accelerate lesion formation through various factors, and it is proposed to use acrolein eliminators as a new target for the long-term improvement of symptoms in patients with neurodegenerative diseases. Their work underscores the importance of understanding the byproducts of food processing and their potential health implications. This research highlights the need for innovative protective strategies to mitigate the formation of such harmful compounds.

Contribution 2 and 3 both focused on plant-based meat analogs and commercial sausages, respectively, examining the content of advanced glycation end products (AGEs) and other potentially harmful substances. In the study by Fu et al., the contents of N^ε^-(carboxymethyl) lysine (CML), N^ε^-(carboxyethyl) lysine (CEL), acrylamide, and key nutritional components in 15 commercial-sold plant-based meat analogs (PBMAs) were determined. The study demonstrated that most PBMAs contain sufficient levels of essential amino acids to satisfy adult nutritional requirements, with the exception of methionine+cysteine, which was identified as their primary limiting amino acid. Notably, a correlation analysis showed that proteins and the profiles of amino acid and fatty acid had little influence on CML but significant influence on CEL and acrylamide. These mechanistic insights provide actionable guidance for mitigating hazardous compounds during PBMA production. Furthermore, in the study by Wang et al., it analyzed the concentrations of AGEs, N-nitrosamines (NAs), α-dicarbonyl, and the proximate composition in two kinds of commercial sausages (fermented sausages and cooked sausages) in the Chinese market. The results showed that due to different processing techniques and additives, there were significant differences in the protein/fat content and pH/TBARS values between fermented sausages and cooked sausages, and both contained some hazardous compounds. It also revealed that AGEs formation was primarily associated with lipid oxidation in fermented sausages and the Maillard reaction in cooked sausages. These results provided a comprehensive perspective on occurrence of NAs and AGEs in sausages. Their findings draw attention to the nutritional composition and safety of these increasingly popular food alternatives, emphasizing the need for careful monitoring of food quality during production.

Contribution 4 presented a mitigation strategy for AGEs and 5-hydroxymethylfurfural (HMF) in butter cookies using natural antioxidants and hydrocolloids. This study selected two dietary natural antioxidants (catechins and curcumin) and two dietary hydrocolloids (chitosan and pectin) for evaluation. The results indicated that catechins had appreciable inhibitory effects on AGEs and HMF formation, while the inhibitory effects of pectin and chitosan were limited. A sensory analysis indicated that catechins and curcumin can reduce the color and taste acceptance of biscuits, while chitosan effectively improves the related sensory characteristics. Consequently, strategic integration of natural antioxidants and hydrocolloids emerges as a viable approach to reconcile the competing demands of sensory acceptability and health-promoting attributes. This study not only contributes to the development of healthier food products but also demonstrates the potential of natural additives to improve food safety.

Contribution 5 and 6 addressed the presence of nitrate/nitrite in leafy greens and the exposure to heavy metals and microplastic-like particles from bivalve consumption, respectively. In the study by Iammarino et al., the nitrite and nitrate levels that characterize Swiss chard and wild rocket samples, as collected on the market, were investigated by using ion chromatography with conductivity detection methods. The findings revealed concerning nitrite/nitrate accumulation in select samples, including instances approaching or surpassing current regulatory thresholds. The study confirmed the need for official control, suggesting the introduction of legal limits for nitrate in Swiss chard and nitrite in both Swiss chard and wild rocket. In the study by Tanaviyutpakdee and Karnpanit, the levels of Pb, Cd, and MP-like particles in bivalves collected from Thailand were determined. The results showed that none of the estimated exposure scenarios to Cd and Pb exceeded the relevant safety levels. However, the Cd intake in children estimated from the worst-case scenario suggested a potential health risk, which provided references for the establishment of targeted food regulations and evidence-based consumption guidelines. These studies remind us of the environmental contaminants that can find their way into our food supply, necessitating vigilant monitoring and risk assessment.

Contribution 7 developed a method for the detection of dichloroanilines and phthalates in rice, while Contribution 8 and 9 focused on the determination of anthraquinones in *Polygonum multiflorum* and aristolochic acid analogs in *Houttuynia cordata*, respectively. In the study by Tsochatzis et al., the ultra-high-performance reversed-phase liquid chromatography–tandem mass spectrometry (UHPLC-MS/MS) method was developed to quantify the two dichloroanilines and six phthalates simultaneously. The developed and validated method provided a quick sample preparation procedure, low qualitative and quantitative detection limits, good chromatographic separation, accuracy, recovery, precision, and non-significant matrix effect. Thus, it provides an easy and fast approach for the quantification of these selected pollutants in rice. In the study by Xu et al., a rapid and effective ultra-high-performance liquid chromatography (UHPLC) method was developed for the determination of five anthraquinones (emodin, physcion, aloe-emodin, rhein, and chrysophanol) in *Polygonum multiflorum*. Further studies demonstrated that this method could be effectively applied to the analysis of five anthraquinones in *Polygonum multiflorum*. And in the study by Yu et al., a new method, which displayed good accuracy, repeatability, and precision, was developed for the simultaneous determination of analogs of aristolochic acids (aristolochic acid I, aristolochic acid II, aristolactam I, and aristolactam AII) in *Houttuynia cordata* via ultra-high-performance liquid chromatography–quadrupole/time-of-flight mass spectrometry (UHPLC–Q/TOF-MS). In addition, the work further confirmed that Houttuynia cordata does not contain carcinogenic substances (AA-I, AA-II, and AL-I), and it should serve as a valuable reference for further safety assessments of *Houttuynia cordata*. These analytical advancements are crucial for the accurate quantification of toxins, enabling more effective food safety evaluations.

Contribution 10 and 11 explored the reduction in furan in coffee and the effects of frying conditions on the content of 3-monochloropropane-1,2-diol esters (3-MCPDE) and glycidyl esters (GE) in palm oil (PO), respectively. In the study by Wang et al., the degradation of the furan content in ground coffee, Maillard model system, and not-from-concentrate (NFC) apple juice by red-fleshed apple anthocyanin extract (RAAE) was studied. The results revealed that RAAEs have a decreasing effect on the furan content in the three systems in different degrees. Thus, the study verified that the antioxidant RAAE, characterized by high safety and low production costs, serves as a viable strategy for furan suppression in thermal-processed foods. In the study by Zhang et al., they selected three experimental parameters for investigation: food type (potato chips and chicken breast), frying frequency, and frying temperature. The findings demonstrated that the formation of 3-MCPDE was affected by the NaCl content, temperature, and time in palm oil during frying. In addition, the formation’s kinetic equations indicated that 3-MCPDE and GE followed zero-order reactions in PO, which offers a theoretical basis for the efficient inhibition of 3-MCPDE and GE in foods. Their work highlights the role of food processing in the formation of contaminants and the potential for process optimization to enhance food quality.

Contribution 12 and 13 investigated the degradation of mycotoxins and the effects of endogenous antioxidants on the formation of chloropropane esters during thermal processing. In the study by Zheng et al., cold plasma as a non-thermal treatment technology was utilized to degrade zearalenone. The data indicated that the zearalenone degradation percentage, influenced by treatment time and voltage, is attributed to reactive species (ozone, free radicals, and ROS) generated in cold plasma. This study provided a theoretical basis for the degradation of zearalenone to a certain extent. And in the study by Liu et al., the effects of endogenous antioxidants (α-tocopherol, stigmasterol, and squalene) on the formation of 2-Monochloropropane-1, 3-diol (2-MCPD) esters, and 3-monochloropropane-1,2-diol (3-MCPD) esters in a model thermal processing of camellia oil were investigated. The findings investigated that these antioxidants presented both prompting and inhibiting effects on the formation of 2- and 3-MCPD esters, and this specific effect is related to the type and content of antioxidant substances. Moreover, the EPR results revealed that CCl_3_·, lipid alkoxyl, N_3_·, and SO_3_· radicals formed during camellia oil processing potentially mediate chloropropanol ester formation, thereby providing insights into the generation mechanism of MCPD esters. These studies contribute to the understanding of how food processing can both introduce and mitigate chemical contaminants.

## 3. Conclusions

In conclusion, the collective body of research represented by these articles paints a comprehensive picture of the complex interplay between chemical contaminants and food quality. From the identification and quantification of contaminants to the development of mitigation strategies, these studies collectively contribute to the ongoing efforts to ensure the safety and quality of our food supply. As the field of food science continues to evolve, it is imperative that we remain vigilant in our pursuit of knowledge and innovation to protect public health and promote sustainable food systems. This Special Issue underscores that the solutions must be as interconnected as the challenges themselves: combining analytical innovation with mechanistic understanding and translating scientific discovery into policy frameworks that prioritize both safety and sustainability. By fostering collaboration across academia, industry, and regulatory bodies, we can strive toward a future where the global food supply is not only abundant but also a guarantee of health and equity for all.
